# Maintenance therapy for AML after allogeneic HCT

**DOI:** 10.3389/fonc.2022.895771

**Published:** 2022-08-09

**Authors:** Rahul K. Nayak, Yi-Bin Chen

**Affiliations:** ^1^ Department of Medicine, Massachusetts General Hospital, Boston, MA, United States; ^2^ Hematopoietic Cell Transplant and Cell Therapy Program, Massachusetts General Hospital, Boston, MA, United States

**Keywords:** AML, AML – acute myeloid leukaemia, maintenance, relapse, transplant

## Abstract

Allogeneic hematopoietic cell transplant (allo-HCT) for eligible patients with acute myeloid leukemia (AML) in first complete remission is a central treatment paradigm to achieve durable remission. However, disease relapse after allo-HCT remains a significant concern and generally portends a poor prognosis. There is significant interest regarding the role for maintenance therapy after allo-HCT for patients with high risk of relapse, regardless of the presence of measurable residual disease. While there are currently no therapies approved for maintenance therapy for AML after allo-HCT, there are a number of ongoing investigations examining the role of maintenance therapies that include targeted agents against FLT3-ITD or IDH mutations, hypomethylating agents, immunomodulatory therapies and cellular therapies. In this review, we examine the current landscape and future strategies for maintenance therapy for AML after allo-HCT.

## Introduction

Allogeneic hematopoietic cell transplant (allo-HCT) for eligible patients with AML in first complete remission is a central treatment paradigm to achieve durable remission for many patients. However, disease relapse remains the leading cause of long-term failure after allo-HCT, and the prognosis after relapse is dismal. With the advent of less toxic or targeted therapies for AML, significant interest has emerged regarding the role of maintenance therapy after allo-HCT to improve overall outcomes by preventing disease relapse.

## Concept of maintenance therapy

Historically, therapy after allo-HCT was difficult to administer due to the reluctance to cause cytopenias, immunosuppression or toxicity in the period immediately after recovery from the peri-allo-HCT period. With the advent of novel agents aimed at targetable mutations and significantly more tolerable chemotherapeutics, maintenance therapy given after allo-HCT has become a more feasible approach. However, the potential benefits of additional chemotherapeutic agents must be balanced against the costs to patients, specifically myelosuppressive and other adverse side effects/toxicities, impaired engraftment, encouragement of graft versus host disease (GVHD) through immunomodulation and overall impact on quality of life. Currently, the role of maintenance therapy after allo-HCT is debated in the field (1, 2), and a number of clinical trials over the last decade have begun to examine the efficacy and safety of maintenance therapy with specific agents.

Generally, the standard of care for patients after allo-HCT in remission has been close monitoring for relapse with individualized treatment approaches towards management of immunosuppression for each patient based on the perceived risk of relapse. The benefit of this approach is that patients who do not relapse are not encumbered by any effects from additional chemotherapeutics after transplant. However, the obvious downside is that up to 40% of patients with AML experience relapse after allo-HCT, and these patients face an extremely poor prognosis with historic 2-year overall survival (OS) rates of < 20% (3).

As a result, there has been great interest in preventative treatment strategies after allo-HCT in patients with high risk of relapse including pre-emptive and maintenance therapy strategies. Pre-emptive therapy is generally understood as a treatment strategy where patients are monitored closely for evidence of measurable residual disease (MRD). At the time of detectable MRD, patients are started on treatment with the hopes to prevent frank morphologic relapse and subsequent complications. The benefit of this approach is that treatment is targeted to patients where there is evidence of impending relapse; however, a downside of waiting to treat until MRD is detected is that kinetics of the disease may prevent any intervention from being effective. Moreover, MRD testing in AML is nowhere near standard for every patient and, oftentimes, depends on local assays for specific mutations. Alternatively, a maintenance therapy approach, the focus of this review, is when treatment is initiated for patients in remission after transplant regardless of MRD status. Conceptually, the ideal maintenance therapy is convenient for patients to receive, able to be initiated soon after transplant for maximal efficacy, does not impair donor hematopoietic engraftment, and does not induce transplant related adverse effects, specifically GVHD.

## FLT3

Around 20% of patients with AML harbor FMS-like tyrosine kinase 3 internal tandem duplication (FLT3-ITD) mutations which have traditionally portended a high risk of relapse and death despite allo-HCT (4). Not surprisingly, there has been significant interest in using available tyrosine kinase inhibitors (TKI) with activity against FLT3, such as midostaurin, sorafenib, gilteritinib, quizartinib and crenolanib as maintenance therapy after allo-HCT to potentially prevent disease relapse.

We published the first such trial – a phase I trial using sorafenib as maintenance therapy (5) in 22 patients, including 3 with primary refractory disease at time of transplant, with FLT3-ITD AML in remission after allo-HCT. The study demonstrated tolerability and feasibility of sorafenib maintenance therapy of 1 year at a dose of 400mg BID, although some patients required dose reduction/discontinuation due mainly to gastrointestinal toxicities. Notably, of the 19 patients in conventional complete remission at time of transplant, only one patient had relapse over the study period, which was a compellingly favorable signal compared to our historical experience. This study was followed by a retrospective cohort analysis of consecutive patients with FLT3-ITD AML who either received sorafenib maintenance therapy (26 patients, either on the aforementioned trial or off-label by their treating physician) compared to contemporaneous controls (55 patients) who were not treated with any maintenance therapy (6). Despite similar baseline patient characteristics, the study found that sorafenib maintenance was associated with improved OS (81% vs. 61%) and 2-year progression-free survival (PFS) (82% vs. 53%) driven by a significantly lower rate of disease relapse (8% vs. 38%).

Subsequently, two randomized controlled trials have validated the efficacy of maintenance sorafenib for FLT3-ITD AML. The SORMAIN trail was a multicenter double-blind placebo-controlled trial in Germany and Austria that randomized 83 patients to sorafenib maintenance vs. placebo for 2 years after allo-HCT (7). The trial showed maintenance sorafenib had improved 2-year relapse-free survival (RFS) compared to placebo (85% vs. 53%, p=0.002) and OS (90.5% vs. 66.2%, p=0.007). About 1 in 5 patients in the sorafenib arm discontinued treatment due to toxicity compared to 5% in the placebo arm. The sorafenib group had higher acute and/or chronic GVHD (77% vs. 60%) and was associated with more episodes of grade 3 skin toxicity (12%) but otherwise had a similar adverse effect profile compared to placebo.

Xuan et al. recently published a multicenter open-label phase 3 randomized trial of 202 patients in China comparing sorafenib maintenance therapy for only 6 months to no intervention (8). They found the 1-year incidence of relapse to be 7% in the sorafenib arm compared to 25% in the observation arm. This benefit translated into improved 2-year OS (82% vs 68%, P=0.012) and 2-year RFS (79% and 57%) with the use of sorafenib. Sorafenib was typically started 30 days after allo-HCT and 59 out of 100 patients required dose reduction (42), interruption (12), or discontinuation (5) due to adverse effects.

However, limitations of these studies need to be acknowledged. This includes that the SORMAIN trial was prematurely closed due to challenges in recruiting patients (purportedly due to off trial access to FLT3 inhibitor maintenance therapy), and a significant number of patients in the study had to discontinue sorafenib due to toxicity. Xuan et al. only studied sorafenib maintenance for 6 months, and most patients required dose reduction. The biggest issue is that the efficacy of FLT3 maintenance therapy in patients who received FLT3 inhibitors (such as midostaurin) prior to allo-HCT is still unknown; only a minority of patients in both of these studies received FLT3 TKI before allo-HCT. Moreover, there was very limited analysis of the impact of MRD on outcomes and its interaction with the use of maintenance TKI. Lastly, it is unclear what the optimal duration of FLT3 TKI maintenance therapy should be given similar benefits observed in all studies to date.

Midostaurin, which is now approved in combination with induction and consolidation chemotherapy for patients with FLT3 mutated AML, has been studied as a maintenance therapy in a small open-label phase 2 randomized trial. The RADIUS trial randomized 60 patients to midostaurin versus standard of care for up to 1 year after transplant for FLT3-ITD AML (9). This study demonstrated the safety and feasibility of midostaurin given after allo-HCT as only 8 patients had to discontinue due to the adverse effects, mainly gastrointestinal related toxicities. While this study was not powered to detect differences in RFS or OS respectively, there was a trend towards a benefit in the midostaurin arm (18-month RFS of 89% vs. 76%).

While it is presumed the efficacy seen in the sorafenib maintenance trials is driven by on-target effect of FLT3 inhibition, there is evidence that suggests sorafenib may also enhance graft-vs-leukemia activity, perhaps through an autocrine mechanism that increases IL-15 and more active cytotoxic T cells (10). It does remain to be proven if the second generation FLT3 inhibitors, which have a narrower spectrum of activity, are also as efficacious as maintenance agents. The Bone Marrow Transplant Clinical Trials Network (BMT CTN) completed accrual on a phase 3 double-blind placebo-controlled RCT examining the role of gilteritinib as maintenance therapy for FLT3-ITD patients with AML in first remission undergoing allo-HCT (BMT-CTN 1506) (11). This study is designed to also examine the significance of FLT3-ITD based measurable residual disease (MRD) at several time points in terms of predicting disease relapse or illustrating which patients may benefit from maintenance therapy. The trial results are eagerly anticipated and will likely provide substantial guidance on the role and choice of agent for maintenance therapy for FLT3-ITD AML, possibly even leading to regulatory approval. Quizartinib has also been studied in a small phase 1 trial of 13 patients which demonstrated safety and feasibility (12) and is also being studied as part of upfront induction/consolidation and maintenance after transplant for up to 36 months in the on-going QUANTUM-First trial (NCT02668653) (13). Crenolanib is under active investigation in a single arm phase 2 trial (NCT02400255).

Overall, there is mounting evidence of the safety and efficacy of FLT3 targeted maintenance therapy, particularly for the use of sorafenib. Based on the strength of the prospective randomized clinical trials and previous retrospective trials, maintenance FLT3 TKI after allo-HCT has become the standard of care at many centers with the specific choice of TKI dependent upon access and availability (2).

## Isocitrate dehydrogenase

Mutations in either IDH1 or IDH2 are found in approximately 20% of patients with AML, and a recent multicenter retrospective analysis suggested that 2 year relapse rates after allo-HCT are 31% and 25% for IDH1 and IDH2 mutated AML, respectively (14). Enasidenib, a selective IDH2 inhibitor, is approved by the Food and Drug Administration (FDA) for refractory/relapsed IDH2 mutated AML. We recently completed a phase 1 clinical trial (NCT03515512) using enasidenib as maintenance after allo-HCT with initial results showing promising data on the tolerability of enasidenib in this setting (15). Preliminary results were presented on the first 16 treated patients with IDH2-mutated AML and myelodysplastic syndrome (MDS) who initiated enasidenib maintenance therapy 30 to 90 days after allo-HCT for up to twelve 28-day cycles. Overall, 11 patients required temporary dose interruptions or dose reductions from 100mg to 50mg, with one patient discontinuing due to grade 3 bilirubinemia. With a median follow up of 11.7 months, 2 (12.5%) patients experienced relapse, and 15 of 16 remain alive with longer-term results eagerly awaited. Ivosidenib, a selective IDH1 inhibitor, is also under investigation in a phase 1 study (NCT03564821) as maintenance therapy after allo-HCT for IDH1 mutated myeloid neoplasms. This trial has completed accrual with initial results expect in late 2022. Of note, a recent sub-analysis of a single-center retrospective review of 59 patients with IDH1/IDH2 mutated AML who underwent non-myeloablative allo-HCT, found that the 22 patients who received an IDH inhibitor either pre- and/or post-transplant had significantly higher overall survival and trend towards improved relapse free survival (not statistically significant) (16).

Larger prospective studies investigating the efficacy of IDH1/2 inhibitors as maintenance after allo-HCT will be necessary to truly understand the efficacy of using these agents to prevent relapse. Complicating issues include off-trial use due to commercial access, the relatively low incidence of each mutation and common co-mutations including FLT3-ITD.

## HMA

There is considerable interest in using hypomethylating agents (HMA) such as azacitidine and decitabine as maintenance therapy, although there is limited prospective evidence regarding their efficacy in this setting. HMA, which induce global hypomethylation/epigenetic modifications, have likely several mechanisms of anti-leukemic effect including direct leukemic cell cycle control and potential immunomodulatory effects (17, 18). HMA have been approved as induction regimens for patients not expected to tolerate intensive induction, and an oral formulation (CC-486) is approved as a maintenance regimen after chemotherapy for patients who are not candidates for allo-HCT (19).

Not surprisingly, many of the early maintenance studies have been challenging to interpret given small sample sizes, heterogeneous risk of relapse and lack of a prospective control group (20). A larger CALGB/Alliance phase 2 study of patients with MDS/AML (80% MDS) examined the use of subcutaneous azacitidine maintenance therapy initiated between 6 weeks to 90 days after transplant (21). Only 65% of patients were able to start treatment and just 41% of those patients were able to complete all 6 cycles of treatment, raising concerns about feasibility. A single center phase 3 open-label RCT of subcutaneous azacitidine given for 12 cycles of maintenance therapy after allo-HCT in high-risk MDS / AML patients failed to show a significant benefit in RFS or OS (22). Again, only a minority of patients in this study completed the 12 cycles (median duration 4 cycles) for a variety of reasons including relapse, adverse effects, and patient preference. Most recently, a phase 2 open-label multicenter RCT examined the use of recombinant human granulocyte colony-stimulating factor (GCSF) and low dose decitabine after allo-HCT for patients with high-risk AML and found the cumulative incidence of relapse at 2 years to be 15% vs 38% (p < 0.01) in favor of the GCSF-decitabine group with a similar incidence of chronic GVHD (cGVHD) in both arms (23).

The recent development of effective oral azacitidine (CC-486) and decitabine (oral decitabine/cedazuridine) formulations has made this class of agents significantly more attractive and feasible as a post allo-HCT maintenance regimen (24). There are currently several ongoing clinical studies examining the safety and efficacy of oral azacitidine (CC-486) as maintenance therapy. Most notably, a phase 1 study of 30 patients showed an acceptable safety profile for a 14 day treatment cycle (25) and a phase 3 trial (AMADEUS, NCT04173533) is currently underway and actively recruiting patients. We are conducting a phase I trial with oral decitabine / cedazuridine as maintenance after allo-HCT for MDS and CMML (NCT04980404), but trials in AML will sure be soon to follow.

In addition, as other agents have been combined with HMA in the non-transplant setting, there are ongoing maintenance therapy trials investigating azacitidine in combination with other agents such as the bcl-2 inhibitor venetoclax (NCT03613532, NCT04128501, NCT04161885), novel agents such as eprenetapopt (APR-246), which shows promise for TP53 mutated myeloid malignancies (NCT03931291), and in concert with donor lymphocyte infusions.

## Hedgehog

Glasdegib, a small molecule inhibitor of sonic hedgehog (shh), has recently been approved by the FDA as part of induction therapy for patients with AML older than 75 who are unfit for an intensive induction regimen. It was recently studied as maintenance therapy in a single-arm phase 2 pilot study of 31 patients with AML or MDS after allo-HCT (26). Results showed that patients in the study had a 2-year RFS of 32% and OS of 39%. This study was limited by the lack of a control population, but at least compared with historical controls, the investigators did not find evidence of significant benefit with maintenance glasdegib. Importantly, about 1/3 of patients experienced grade 3 or 4 adverse effects (myalgia and gastrointestinal side effects), and the majority of patients required drug interruption or dose reduction.

## Bcl-2

Venetoclax is a selective small molecule inhibitor of the B-cell lymphoma-2 (Bcl-2) protein, a key regulator of apoptosis. A recent phase 1 feasibility trial of 23 patients post allo-HCT (high risk AML and MDS), showed that venetoclax was tolerable for up to 1 year for most patients, although 11% of patients discontinued due to adverse effects or other transplant complications and half required dose interruption and adjustments due to side effects including cytopenias and GI related side-effects (27). As mentioned above, there are several studies that are actively investigating the safety and feasibility of venetoclax in combination with hypomethylating agents, the largest being the VIALE-T trial which is a phase 3 randomized study (NCT04161885).

## Anti-CD33

CD33 is a myeloid differentiation antigen that is highly expressed in AML and is the target of certain antibody drug conjugates such as gemtuzumab ozogamacin (GO). GO has been approved as part of upfront standard induction chemotherapy for CD33^+^ AML, and there has been some interest in its use as post-transplant maintenance therapy. The main concerns of using GO after allo-HCT are the incidence of cytopenias and its known association with hepatic veno-occlusive disease (VOD)/sinusoidal obstruction syndrome (SOS) (28). There has been one small study of 10 patients that used low dose GO [3mg/m (2)] in combination with azacitidine in high-risk AML patients after allo-HCT and showed relative tolerability with no observed cases of VOD/SOS (29). Larger studies will need to be conducted to determine if this is a safe and potentially efficacious approach.

## HDAC inhibitors

Histone deacetylase (HDAC) inhibitors are thought to possess anti-leukemic activity through epigenetic modifications and its theorized immunomodulatory activity may help graft vs. leukemia (GVL) and mitigate GVHD after allo-HCT. A phase 1/2 trial examined the use of panobinostat after allo-HCT for 42 patients with AML or MDS (30). The majority of patients were transplanted with active disease and then started on panobinostat maintenance therapy approximately 3 months after transplant. The estimated 2-year OS was 88% and disease-free survival was 74%. Slightly over half of patients were able to complete 1 year of maintenance therapy with the remainder discontinuing due to adverse effects or relapse. Over half of patients experienced grade 3 or 4 toxicity, such as cytopenias, constitutional symptoms, GI symptoms, and neurological adverse effects, although all fully resolved with interruption of panobinostat. Recently, a German phase 3 trial in high-risk AML (NCT04326764) has completed accrual and results are awaited.

## Other immunomodulators/immunotherapy

The use of lenalidomide for its immunomodulatory properties has been investigated for AML after allo-HCT. The exact mechanism of action is unknown but lenalidomide is thought to promote natural killer and cytotoxic T cell activity as well as inhibit regulatory T cells after transplant. There have been previous safety concerns with lenalidomide inducing GVHD as observed in patients with high-risk multiple myeloma after allo-HCT (31), and a prior prospective phase 2 study of patients with high risk MDS or AML was stopped early due to high rates of GVHD (32). A recent phase 1 feasibility trial of 16 patients was recently published showing tolerability of lenalidomide 10mg, although adverse effects including cytopenias and GI intolerance were common (33). Of note, due to prior concerns that lenalidomide could induce GVHD, this study did not initiate lenalidomide until 6 months after transplant per FDA guidance.

An approach of using azactidine with lenalidomide in 29 patients with relapsed AML/MDS after allo-HCT had relatively low rates of GVHD, where only 3 patients developed GVHD (34). However, patients with a history of significant GVHD were excluded and all patients were recipients of *in vivo* T-cell depleted allografts. These characteristics as well as the setting being therapy for relapsed disease may explain the relatively low rates of lenalidomide induced GVHD that may not translate if lenalidomide is used as an upfront maintenance agent even with concurrent HMA therapy.

While there has been interest in the use of immune checkpoint inhibitors (CPI) for relapsed disease for enhanced GVL effect, there is considerable risk due to potential immune related adverse effects, specifically increased rates of GVHD. A phase 1 trial of 28 patients examining the use of ipilimumab to treat relapsed disease in a variety of hematologic malignancies after allo-HCT found that 5 patients had a complete response and 2 had a partial response. Notably, 4 of the patients with complete response had extramedullary AML (35). However, significant immune-related adverse events, including one death, were observed in 6 patients. This dovetails with findings in lymphoid malignancies, where patients with relapsed disease after allo-HCT were treated with CPI and exhibited increased adverse events including rapid onset of severe and treatment refractory GVHD (36). The use nivolumab as maintenance therapy after consolidation therapy, for patients who are not eligible for allo-HCT has been under investigation for patients with high-risk AML or with MRD (NCT02532231, NCT02275533).

## Cellular therapy

### DLI

For decades, there have been investigations using donor lymphocyte infusion (DLI) to treat patients with relapsed disease. In theory, DLI provides non-tolerant donor T cells which may enhance the GVL effect but at the potential cost of causing GVHD. The best data in support of the use of DLI has been its use as a salvage treatment to induce complete remission for patients with indolent malignancies such as relapsed chronic phase CML. The otherwise limited utility of DLI as primary therapy for frankly relapsed disease, particularly its limitations in the treatment of relapsed AML, has been reviewed elsewhere (37).

Prophylactic DLI as maintenance therapy and pre-emptive DLI in the setting of MRD or mixed chimerism has remained of interest. One prospective study of low-dose prophylactic DLI administered soon after allo-HCT examined 15 patients with AML and acute lymphocytic leukemia (ALL) treated with alemtuzumab-based conditioning (38). While there were no relapses observed, 4 out of 15 patients died from steroid-refractory GVHD leading to termination of the study. However, a retrospective cohort review of patients with high-risk AML and MDS who received up to 3 doses of escalating DLI starting 120 days after allo-HCT, found OS at 7 years of 67% compared with 31% of patients in a “control” arm who did not receive DLI (39). While the study does show feasibility of prophylactic DLI, the major limitations are that it excluded patients from analysis who had any history of GVHD prior to DLI and the “control” cohort of patients was treated at a different institution with differing transplant regimens.

A more recent registry-based survey from the Acute Leukemia Working Party of the European Group for Blood and Marrow Transplantation examined the use of DLI in the preemptive setting for MRD or mixed chimerism or prophylactically for high-risk patients (40). This study found that 6% of patients died from DLI-induced GVHD with patient age, advanced disease at transplantation, shorter time from transplantation, and prior acute GVHD as predictors of DLI-induced GVHD. Perhaps, most interestingly, the preemptive use of DLI for patients with MRD was shown to be able to reduce MRD in the majority of patients.

There is clearly a need for collaborative prospective trials on prophylactic or preemptive DLI after allo-HCT to truly elucidate a treatment effect. Certainly, more homogeneity in the transplant platform and state of immune reconstitution / immunosuppression at time of DLI administration would be preferred. Not surprisingly, there has been interest in investigating the combination of DLI with immunomodulatory agents as maintenance therapy including a recent phase 2 trial investigating prophylactic low dose azacitidine and DLI in high-risk AML/MDS (41).

### Engineered T Cell therapy

While CAR T-cell therapy has emerged as a major modality of therapy for certain B-cell malignancies, there has been limited utility in the treatment of AML, largely due to a lack of AML-specific targets (42). While there are some ongoing investigations of CAR T-cells for the treatment of relapsed/refractory AML, such constructs are not yet being actively investigated as post allo-HCT maintenance therapy. Engineered T-cell receptor (TCR) therapy is another form of cellular therapy actively being studied and may have an attractive role as maintenance therapy after allo-HCT. Prior studies have shown that some patients who received minor H antigen mismatched DLI after relapse and developed expansion of hematopoiesis-restricted minor histocompatibility antigens HA-1 or HA-2-specific T-cells had complete regression of leukemia (43, 44). Taking advantage of this observation, a novel approach to develop engineered TCR therapy with high affinity to HA-1/HA-2 has been recently demonstrated (45), and a trial of donor-derived TCR therapy after haploidentical allo-HCT is planned to open in 2022. In addition, a phase 1 trial recently demonstrated the safety of donor derived T cells which were activated and expanded against AML-related antigens (PRAME, WT1, Survivin, and NY-ESO-1) in 25 patients (17 at high risk of relapse and 8 patients with relapsed disease). There were no significant adverse effects such as GVHD noted in this small trial and some *in vivo* anti-leukemic effects were noted in the relapsed disease cohort with 2 patients showing partial or complete response.

### NK cells

Natural killer (NK) cells are thought to play a role in the immunologic control of AML and the GVL response after allo-HCT, and there is growing interest in adoptive NK cell-based therapy to prevent relapse after transplant. After transplantation, NK cells are the earliest lymphocytes to engraft, and there is evidence that high donor NK cell chimerism at 14 days is associated with lower relapse rate. In addition, it has long been observed that donor NK cell alloreactivity mediated through killer-cell immunoglobulin-like receptors (KIRs) ligand (e.g., HLA class 1 incompatibility) has also been associated with reduced risk of relapse and increased survival (46), though this has not always been consistently observed (47). Additionally, there is evidence that donor NK cells with certain haplotypes of KIR genes with multiple activating KIRs, such as the “B-haplotype,” are associated with reduced risk of relapse likely mediated though a lower threshold for donor NK cell cytotoxic effector function (48). These observations have led to interest in whether adoptive NK cell therapy after transplant could augment the GVL effect.

Active trials investigating adoptive NK cell-based therapy have broadly been characterized as trials of NK cell enriched DLI and the administration of cytokine-induced activated NK cells. One recent phase 1/2 study of 25 patients with myeloid malignancies undergoing haploidentical transplant examined the safety and feasibility of ex-vivo expansion of donor derived NK cells which was then delivered on days -2, +7 and +28 (49). This study, compared with 160 matched historical controls, found a 2-year relapse rate of 4% vs. 38% (p=0.014) and disease free survival of 66% vs. 44% (p=0.01) in favor of those receiving NK cell therapy. Notably, most patients did not receive KIR-ligand-mismatched or KIR B-haplotype donors. Another approach that has been trialed was ex vivo activation of NK cells. A recent phase 1 study administered prophylactic IL-2 activated NK cells in 16 patients after allo-HCT for a variety of hematologic malignancies (50). Overall the treatment was well tolerated with no significant dose limiting toxicities or adverse events noted in the first 30 days. Rates of cGVHD were similar to historical expectations, with 3 patients developing moderate/severe cGVHD that responded to steroid treatment. Another study has recently examined the use of IL-12, IL-15, and IL-18 to create cytokine-induced memory like (CIML) NK cells (51). A phase 1 study examined the use of these CIML NK cells in 2 patients with AML with relapsed disease after transplant and found evidence of treatment response and no evidence of leukemic blasts on +28 bone marrow biopsy. CAR-NK therapy, similar to CAR-T therapy, has likewise been limited by lack of AML-specific targets.

### Vaccines

Vaccines that prime the immune system against leukemic cells have been an area of active interest for patients with active disease and in remission. Both peptide-based and whole tumor cell-based vaccines have been developed for AML (52), although only a few studies have looked at vaccines as a form of maintenance therapy. The theoretical benefit of peptide-based vaccines is feasibility, tumor selectivity, and minimizing the potential risk of GVHD. However, as there is no specific or universal antigen for AML, prior strategies have targeted proteins overexpressed in AML, such as Wilms’ tumor 1 (WT1) and PR1 (52). A phase 1 study of a WT1 vaccine in 9 high-risk patients (5 with AML) after allo-HCT was conducted that generally demonstrated safety with no significant adverse events noted for most patients (53). Interpretation of efficacy was obviously challenging given the small sample size with biological heterogeneity of the underlying AML although all 4 AML patients in complete remission at time of allo-HCT remained in complete remission at over 2 years after vaccination.

Alternative vaccine approaches that use whole tumor cells as a source of antigens have also been explored. This includes gene-transduced tumor cell vaccine (GVAX), where leukemic cells are manipulated to express GM-CSF, and dendritic cell/AML fusion vaccines, where donor-derived dendritic cells are fused with patient-derived AML cells. A phase 1 study examining GVAX for 15 patients with AML/MDS demonstrated safety of the approach with comparable rates of GVHD compared to a historical control with a 2-year Kaplan-Meier estimate of OS of 57%. Results from a phase 2 trial were recently published by the same group, which randomized 57 patients with AML/MDS to GVAX versus placebo after allo-HCT. GVAX was well tolerated with only injection site reactions noted as adverse events; however, no difference in overall survival or progression free survival were observed in GVAX recipients compared to placebo (54). Results from a phase 1 trial of a donor derived dendritic cell/AML fusion vaccine were also recently published (55). This study enrolled 17 patients with AML after transplant, of which 11 participants were able to have vaccine successfully generated and administered. Promisingly, of patients who received the vaccine, 10 remained in complete remission at a median time of 21 months after allo-HCT.

## Discussion

Over the last decade, there has been considerable interest in the use of maintenance therapy for patients with AML after allo-HCT to potentially reduce the significant risk of disease relapse. **Table 1** lists the major ongoing clinical trials in this space. The agents chosen are often repurposing therapies with proven efficacy in other settings due to familiarity as well as commercial access. There have been only a handful of prospective phase 2 or 3 studies powered to examine the efficacy of specific maintenance therapies that have completed or are currently still accruing patients (**Table 2**).

**Table 1:** Select Active Clinical Trials for Maintenance Therapy after Allo-HCT for patients with AML.

Drug Class/Intervention	Description	Duration of maintenance therapy	Status	Clinical Trial Identifier
Gilteritinib	Phase 3 double-blind, placebo RCT in FLT3-ITD AML	Up to 2 years maintenance	Completed accrual, 356 participants	NCT02997202 (BMT-CTN 1506)
Quizartinib	Phase 3, double-blind, placebo RCT (upfront and as maintenance) in FLT3-ITD AML	36 months of treatment	Completed accrual, 539 participants	NCT02668653 (QUANTUM-First)
Crenolanib	Phase 2, open label/single arm in FLT3+ AML	Up to 2 years maintenance	Completed accrual, 48 participants	NCT02400255
Enasidenib	Phase 1, open label in AML/MDS/CMML	Up to 12 months	Completed accrual w/initial results, 16 participants	NCT03515512
Ivosidenib	Phase 1 open label in AML/MDS/CMML	Up to 12 months	Completed accrual, 18 participants, initial results expected in late 2022	NCT03564821
Oral azacitidine	Phase 3, double-blind, placebo RCT in AML/MDS	Up to 12 months	Recruiting, estimated enrollment 324 participants	NCT04173533
Oral decitabine/cedazuridine	Phase 1, open label in MDS/CMML	Up to 2 years	Recruiting, estimated enrollment 22 participants	NCT04980404
Azacitidine + Venetoclax	Phase 1, open label in high risk AML/MDS/MPN overlap	Up to 12 months	Recruiting, estimated enrollment 68 participants	NCT03613532
Azacitidine + Venetoclax	Phase 2, open label trial in AML and other hematologic malignancies	Up to 12 months	Recruiting, estimated enrollment 125 participants	NCT04128501
Azacitidine + Venetoclax	Phase 3, open label RCT in AML	Up to 24 months	Recruiting, estimated enrollment 424 participants	NCT04161885 (VIALE-T)
Azacitidine + eprenetapopt	Phase 2, open label trial in TP3 mutated AML/MDS	Up to 12 months	Completed recruitment, 33 participants	NCT03931291
Panobinostat	Phase 3, open label RCT in AML/MDS	Unclear but at least 1 year	Completed recruitment, 52 participants	NCT04326764
DC/AML fusion cell vaccine	Phase 1 – 2 vaccines, 3 weeks apart, +/- decitabine	2 vaccines	Recruiting, estimated enrollment 45 participants	NCT03679650

**Table 2:** Select published prospective or randomized trials with >50 participants for maintenance therapy post allo-HCT.

Drug/Intervention	Description	Findings	Comment	Clinical Trial Identifier
Sorafenib	Phase 2, double-blind placebo RCT of 83 patients with FLT3-ITD AML. Intervention: Sorafenib 400mg BID vs. placebo for 2 years. Sites: Germany/Austria	2-year RFS: 85% vs. 53% (p=0.002) 2-year OS: 90.5% vs. 66.2% (P=0.007)	- All adults - Maintenance starting day +60 to +100 -AEs: Increased rates of skin toxicities, renal/electrolytes issues, and cGVHD. - No FLT3-TKI prior to HCT - Closed early due to poor accrual	DRKS00000591 (SORMAIN)
Sorafenib	Phase 3, open label RCT of 202 patients with FLT3-ITD AML. Intervention: Sorafenib 400mg BID versus SOC for 6 months Sites: China.	1-year relapse rate: 7% vs. 24.5% (P=0.001) 2-year RFS: 78.9% vs. 56.6% (P<0.0001) 2-year OS: 82.1% vs. 68.0% (P=0.012)	- Adults 18-60 years - Maintenance starting day +30 to +60 - 30% MRD at time of transplant, 10% MRD at time of enrollment - AEs: Skin, cytopenia - No significant difference in GVHD - No FLT3-TKI prior to HCT	NCT02474290
Midostaurin	Phase 2, open label RCT of 60 patients with FLT3-ITD AML. Intervention: Midostaurin 50mg BID versus SOC for up to 1 year. Sites: US and Canada.	18 month RFS: 89% vs. 75% (P = 0.27)	- Adults 18-70 years - Up to 12 cycles, only 50% in each group completed all cycles - AEs: Increased GI symptoms (diarrhea, nausea, vomiting) - Similar rates of GVHD - Not powered to look at efficacy	NCT01883362 (RADIUS)
Azacitidine	Phase 2, single arm trial of azacitidine in 63 patients with MDS/AML. Intervention: Azacitidine 32 mg/m^2^ SQ x5 days per 28 day cycle for up six cycles (single arm). Sites: US	Cumulative relapse at 2 years: 25% 2-year OS: 45.7% Median OS: 1.6 years	- Adults 60-75 years - All patients received RIC - Maintenance started day +42 to +90 - 80% of patients with MDS - 65% were able to start treatment and 41% got 6 cycles of treatment	CALGB 100801 (Alliance)
Azacitidine	Phase 3, open label RCT of 187 patients with in high risk AML/MDS Intervention: Azacitidine 32 mg/m^2^ SQ x5 days per 28 day cycle for up to 12 cycles vs. SOC. Sites: MD Anderson (US)	Median RFS: 2.07 years vs. 1.28 years (P = 0.43) Median OS: 2.52 years vs. 2.56 years (P=0.85)	- Adults 18-75 years - Treat arm on average (median) only 4 cycles received - AEs: Myelosuppression - Single center	NCT00887068

RCT, Randomized Controlled Trial; RFS, relapse free survival; OS, overall survival; MRD, measurable residual disease; AE, adverse effect; SOC, standard of care; SQ, subcutaneous.

The challenges in conducting clinical trials in the allo-HCT maintenance setting are numerous. First, patients have significant competing risks including cytopenias, conditioning regimen induced organ toxicity, opportunistic infection and GVHD. Second, while drugs may be approved in other contexts with well understood side effects, the adverse effects may be more difficult to predict in the post-transplant immunological environment, including the potential impact on GVHD, which necessitates first conducting thoughtful safety studies. Third, recruitment of patients for treatments with the potential for significant adverse effects may be challenging when there is no active disease. Lastly, for therapies that have been approved for other indications and there is off-label commercial access, there may be significant off-study use with patients and treating physicians being reluctant to participate in a randomized controlled trial.

Currently, the only randomized clinical trial data supporting the use of maintenance therapy is for FLT3 inhibitors in patients with FLT3-ITD AML. The recent publication of two randomized trials that demonstrated the significant benefit in RFS and OS for sorafenib maintenance therapy for FLT3-ITD AML has generated significant excitement in the field. Outside of FLT3-ITD AML, there appears to be only limited phase 3 trials in progress. Most notably, the results of the AMADEUS trial, examining oral azacitidine (CC-486), will be of significant interest to follow. Perhaps surprisingly, while CC-486 has been FDA approved for maintenance therapy for patients who are not candidates for allo-HCT, the larger clinical trials studying HMA maintenance post allo-HCT have not demonstrated significant benefits to date. The use of oral therapy should hopefully improve adherence and tolerability, which seems to have been a major limitation of prior trials where a low proportion of patients actually completed the treatment per protocol for various reasons. In addition, results from a German phase 3 trial investigating the HDAC inhibitor panobinostat are eagerly anticipated. Given the potential immunomodulatory effects with both HMA and HDAC inhibitors, attention to adverse events such as GVHD will be important to follow in addition to effects on disease relapse.

As more specifically targeted agents for AML are developed, trials of maintenance therapy targeting genomically defined subsets will only increase. To truly define the efficacy of such approaches, large collaborative efforts will be essential given the relatively low incidence of such mutations. For example, investigation on the use of IDH1/2 inhibitors as maintenance therapy for IDH1/2 mutated AML remains in very early stages with only phase 1 trials to date. While both enasidenib and ivosidenib appear to be well tolerated in the post allo-HCT setting, larger randomized trials designed to assess efficacy are sorely needed.

While not explored in detail in this review, it should be mentioned that disease-specific serial MRD testing will play an increasingly important role in stratifying patients for the highest risk of relapse (56–59). Utilizing MRD status to more precisely target patients who would most benefit holds promise for limiting the toxicity of post allo-HCT therapy. In addition, serial MRD measurements can be used to monitor treatment response while on therapy and potentially serve as surrogate endpoints. However, pitfalls of overutilizing MRD presently include heterogeneity of mutations, some of which do not represent leukemia driving mutations, and the fact that certain MRD measures have not been definitively proven to imply imminent relapse (60). Maintenance therapy trials that prospectively plan to follow MRD status between intervention and control arms will be invaluable to determine the clinical utility of MRD.

## Conclusion

Allo-HCT for eligible patients with acute myeloid leukemia in first complete remission remains a central treatment paradigm to achieve sustained remission. Nevertheless, relapse after transplant remains the leading cause of long-term failure. Over the last decade, intense interest has emerged as to whether maintenance therapy after allo-HCT can reduce relapse and improve post-transplant survival. Here, we have reviewed the principles of this treatment paradigm and the most recent advances including ongoing trials. The most promising data supporting maintenance therapy is for FLT3-TKI after allo-HCT for patients with FLT3-ITD AML and this has become the standard of care at many institutions. There are numerous other promising agents, such as other genomically targeted agents, HMA, immunomodulatory therapies, and cellular therapies, although well-designed clinical trials need to be conducted. Toxicity (chemotherapeutic cytotoxicity vs. immunologic cytotoxicity/GVHD) is the limiting factor for many of these approaches, as many treatments are repurposed therapies from the relapsed/refractory disease setting and may not be acceptable for patients without active disease. In the coming years, clinical trial results for gilteritinib, IDH inhibitors, oral azacitidine, azacitidine and venetoclax, panobinostat, and various cellular therapy approaches are expected to greatly improve our understanding of the role of maintenance therapy for AML after allo-HCT.

## Author contributions

RN and Y-BC conceived of the topic, designed and wrote the manuscript. All authors contributed to the article and approved the submitted version.

## Conflict of interest

YBC has served as a consultant for Novartis, Incyte, Jasper, Celularity, Equilium, Actinium, Daiichi and Abbvie.

The remaining author declares that the research was conducted in the absence of any commercial or financial relationships that could be constructed as a potential conflict of interest.

## Publisher’s note

All claims expressed in this article are solely those of the authors and do not necessarily represent those of their affiliated organizations, or those of the publisher, the editors and the reviewers. Any product that may be evaluated in this article, or claim that may be made by its manufacturer, is not guaranteed or endorsed by the publisher.
